# Leptomeningeal Enhancement due to Neurosarcoidosis Mimicking Malignancy

**DOI:** 10.1155/2020/9513576

**Published:** 2020-04-20

**Authors:** Sahara N. Saltijeral, Horiana B. Grosu, Henriette De La Garza, Barbara O'Brien, Gloria Iliescu

**Affiliations:** ^1^Departments of Internal Medicine, Instituto Tecnologico y de Estudios Superiores de Monterrey, Monterrey, Mexico; ^2^Departments of Pulmonary Medicine, The University of Texas MD Anderson Cancer Center, Houston, TX, USA; ^3^Departments of Neuro-Oncology, The University of Texas MD Anderson Cancer Center, Houston, TX, USA; ^4^Internal Medicine The University of Texas MD Anderson Cancer Center, Houston, TX, USA

## Abstract

The present report describes the case of a 56-year-old African American man experiencing progressive disequilibrium, lower extremity weakness, difficulty walking, and hearing loss. Brain magnetic resonance imaging showed leptomeningeal enhancement. Initial differential diagnosis was broad, including malignant, infectious, and inflammatory etiologies. The cerebrospinal fluid analyses demonstrated lymphocytic pleocytosis, hypoglycorrhachia, and hyperproteinorrachia but no other abnormalities. An extensive infectious disease workup was negative. Positron emission tomography revealed hypermetabolic lymph nodes in the right mediastinum and right hilum, correlating with findings on endobronchial ultrasonography. Subsequently, image-guided fine-needle aspiration of the right upper paratracheal lymph node was performed, and biopsy studies showed noncaseating granulomatous inflammation. Based on the clinical picture, the diagnosis of neurosarcoidosis was made, and high-dose steroids were started and resulted in significant improvement.

## 1. Introduction

Sarcoidosis is an idiopathic, noninfectious inflammatory disorder characterized by formation of noncaseating granulomas [1, 2]. The annual incidence is highest among African Americans, with 39.1 and 29.8 cases per 100,000 in females and males, respectively [3]. Imaging findings suggestive of neurosarcoidosis are found in 10% of patients with systemic sarcoidosis, and among these patients, only 5% develop symptoms [1, 3, 4]. Meningeal disease, which occurs in 10% to 20% of patients with neurosarcoidosis, usually presents as aseptic meningitis [5]. Meningeal disease in sarcoidosis may present as abnormal leptomeningeal enhancement, meningeal mass lesion, or dural mass lesion on MRI examination. In addition, meningeal sarcoidosis presenting as a mass on imaging can mimic intracranial tumors such as meningiomas. The case presented here, in which meningeal neurosarcoidosis mimicked malignancy, illustrates the diagnosis of this rare condition.

## 2. Case Report

A 56-year-old African American man presented complaining of progressive disequilibrium, lower extremity weakness, difficulty walking, and hearing loss over a 2-year period. On physical examination, he was found to have a mildly unsteady casual tandem gait. There were no cranial nerve deficits aside from slightly decreased hearing according to a bilateral finger rub test. Mildly decreased pinprick sensation was seen in the bilateral distal lower extremities. The patient's bilateral hip flexion/extension was graded 4/5; otherwise, his strength was graded 5/5 in all 4 extremities. His symmetric reflexes were graded 3/4 in all extremities. Neither magnetic resonance imaging (MRI) nor computed tomography revealed abnormal findings at initial presentation. A lumbar puncture was performed, and the cerebrospinal fluid (CSF) analyses demonstrated lymphocytic pleocytosis, hypoglycorrhachia, and hyperproteinorrachia but no other abnormalities. Infectious disease workup was negative.

**Figure 1:**
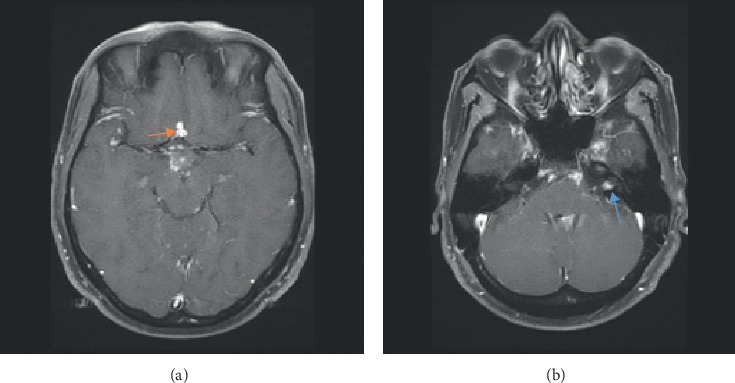
(a) MRI brain, axial depicting nodular enhancement, a low frontal lesion close to the anterior communicating artery lobe (orange arrow). (b) Nodular enhancement of the left internal auditory canal (blue arrow).

**Figure 2:**
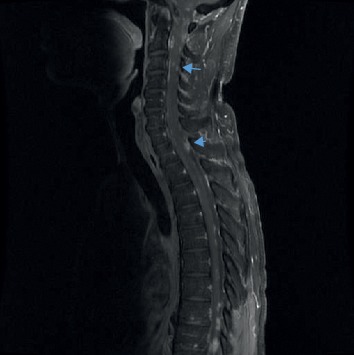
MRI spinal cord, sagittal, T2-weighted showing hypointense nodules localizing to the level of the inferior endplate of T2 and C7 (blue arrows).

**Figure 3:**
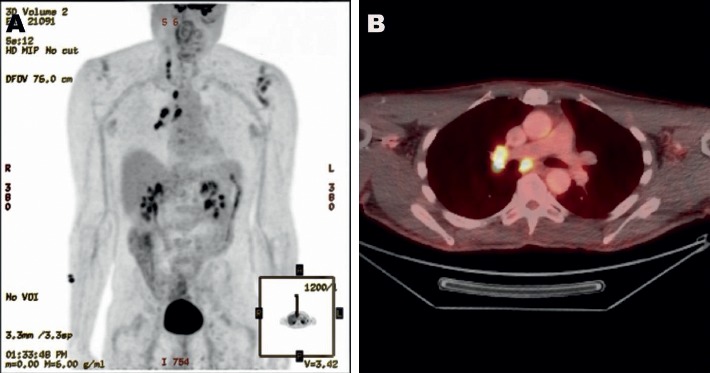
(a) Contrast-enhanced FDG-PET showing hypermetabolic lymph nodes in the right upper and lower paratracheal nodes, right hilar nodes, and subcarinal nodes. (b) Hypermetabolic subcarinal and right hilar lymph nodes.

**Figure 4:**
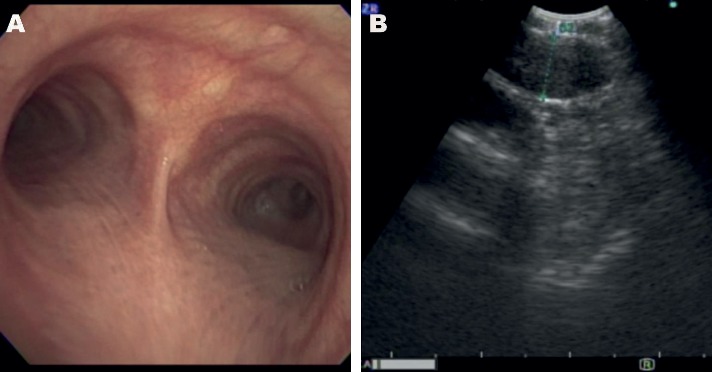
(a) Image of main carina showing normal mucosa (blue arrow:right mainstem bronchus and orange arrow:left mainstem bronchus). (b) EBUS image of the right upper paratracheal lymph node measured with green annotation.

Later, the patient's symptoms progressed, and repeat brain MRI performed two months after his initial presentation showed leptomeningeal enhancement that raised concerns of malignancy; however, infectious and inflammatory etiologies were in the differential as well. Subsequent brain MRI performed two months later exhibited nodular focal leptomeningeal enhancement overlying the superior cerebellum, interpeduncular cistern, frontal region, superior temporal lobe, periventricular region, near the obex, and in the left internal auditory canal (Figures 1(a) and 1(b)). Spine MRI showed diffuse multilevel nodular and plaque-like areas of enhancement along the surface of the cervical cord (Figure 2). Positron emission tomography (PET) showed hypermetabolic lymph nodes in the right upper and lower paratracheal lymph nodes, right hilum, and subcarinal lymph nodes (Figures 3(a) and 3(b)). Subsequent CSF analyses revealed leukocytosis, hypoglycorrhachia, hyperproteinorrachia, and angiotensin-converting enzyme (ACE, 8.6 U/L). Endobronchial ultrasonography showed enlarged lymph nodes corresponding to the PET imaging (Figures 4(a) and 4(b)). Image-guided fine-needle aspiration of the right upper paratracheal lymph node was performed subsequently, and the biopsy analyses showed noncaseating granulomatous inflammation.

Neurologic examination performed three months after the patient's initial presentation showed clinical decline with mild cognitive difficulties, right foot weakness, decreased pinprick sensation in the feet with preserved vibratory sense, proprioception without alterations, and hyperreflexia. The patient developed sensory stocking-glove distribution neuropathy and increasing gait unsteadiness. Spine and brain MRI performed at this time showed worsening of the leptomeningeal enhancement.

On the basis of the workup, the patient was diagnosed with probable neurosarcoidosis, and high-dose steroids were started. At a follow-up visit one month after the start of steroid treatment, the patient had responded adequately; in particular, his strength and gait had notably improved. Subsequent spine and brain MRI showed near-complete resolution of the nodular leptomeningeal enhancement.

## 3. Discussion

Sarcoidosis can affect any organ system and present in virtually any manner [6]. The lungs or thoracic lymph nodes are involved in up to 90% of patients. Although only 10% of patients with systemic sarcoidosis have imaging findings suggestive of neurosarcoidosis—and few of these patients develop symptoms [3]—autopsy studies found central nervous system involvement in 15% to 20% of systemic sarcoidosis cases [5]. These postmortem studies have advanced the understanding of the pathogenesis of neurosarcoidosis, demonstrating that granulomatous dissemination can follow hematogenous or lymphatic routes as well as local extension. Presumably, hematogenous dissemination is the preferred route in the central nervous system [5]. Because brain neurosarcoidosis usually presents as an extensive variety of nonspecific symptoms, exclusion of other diagnoses is mandatory [3].

In a prospective study by Kidd [7], from a total of 166 patients with a “highly probable” diagnosis of neurosarcoidosis according to the World Association of Sarcoidosis and Other Granulomatous Diseases sarcoidosis organ assessment instrument, 67 patients presented involvement of leptomeninges and 42 patients presented with encephalopathy with headache, drowsiness, and cognitive slowing. Of these 42 patients, 17 had seizures and 8 had hydrocephalus and a brainstem syndrome with ataxia of gait, diplopia, and vertigo. In the same study, lesions in the spinal cord were seen in 28 patients.

Sarcoidosis is diagnosed on the basis of compatible findings from clinical examination and imaging studies, and the diagnosis is reinforced by histologic findings of noncaseating epithelioid-cell granulomas, in at least 1 organ, in the absence of organisms or particles [8]. Meningeal involvement in neurosarcoidosis can appear as nodular or diffuse leptomeningeal enhancement on contrast-enhanced T1-weighted MRI [2]. An important part of the initial CSF examination is to rule out infections, central nervous system disease, and malignancy [3]. Our patient went through an extensive infectious disease workup, including fungal, viral, and bacterial screens to exclude other compatible diagnoses. In patients with neurosarcoidosis, CSF examination generally exhibits a picture of chronic lymphocytic meningitis with lymphocyte predominance, hyperproteinorrachia, and hypoglycorrhachia. [4] Our patient's CSF analyses showed 42 C/mcL white blood cells, glucose <30 mg/dl, proteins >600 mg, and an ACE level of 8.6 unit/L. A high level of ACE in the CSF has a low sensitivity (between 24% and 55%) but high specificity (around 94%) for neurosarcoidosis and therefore may raise suspicion of the disease [2]. FDG-PET can reveal areas of hypermetabolism that correspond to active lesions in asymptomatic sites, which can be used to identify suitable sites to perform a biopsy [2]. Our patient's PET demonstrated hypermetabolic right paratracheal lymph nodes, from which the biopsy was taken. Histopathologically, neurosarcoidosis is characterized by the presence of noncaseating epithelioid granulomas, the same lesions as those found elsewhere in the body in cases of systemic disease [2, 3]. Our patient's biopsy showed anthracotic pigmented-laden macrophages and aggregates of epithelioid histocytes, suggestive of noncaseating granulomatous inflammation. Given the clinical picture, the probable diagnosis was made.

Treatment of neurosarcoidosis is based on general consensus, given that there are no established guidelines [2]. Corticosteroids are a mainstay in the treatment of neurosarcoidosis and are used as first-line agents unless severely contraindicated [4]. Our patient responded satisfactorily to high-dose steroids, resulting in improvement of radiological abnormalities.

## Abbreviations

ACE: Angiotensin-converting enzyme
CSF: Cerebrospinal fluid
FDG: Fluorodeoxyglucose
PET: Positron emission tomography
MRI: Magnetic resonance imaging.
